# The stress-fascia-pain axis in myofascial pain syndrome: a mechanistic integrative review and conceptual synthesis

**DOI:** 10.3389/fpain.2026.1834354

**Published:** 2026-08-06

**Authors:** Ali Gür

**Affiliations:** Department of Physical Medicine and Rehabilitation, Algology, Faculty of Medicine, Gaziantep University, Gaziantep, Türkiye

**Keywords:** autonomic nervous system, central sensitization, fascia, HPA axis, myofascial pain syndrome, stress, systematic mapping review, trigger points

## Abstract

**Background:**

Myofascial pain syndrome (MPS) is common in musculoskeletal practice, but its persistence, recurrence, and clinical heterogeneity are not fully explained by a muscle-only model. The integrated hypothesis remains central to understanding the active trigger point itself, yet patients with longstanding symptoms often show a broader picture, including stress-related fluctuation, disturbed sleep, regional spread, autonomic imbalance, and only partial benefit from strictly local treatment.

**Objective:**

To examine whether trigger-point biochemistry, fascial dysfunction, stress-related neuroendocrine change, autonomic imbalance, and altered pain processing can be brought together within a single account of persistence and recurrence in MPS.

**Methods:**

We conducted a mechanistic integrative review using PRISMA 2020 reporting principles. MEDLINE/PubMed, Scopus, Web of Science Core Collection, and the Cochrane Library were searched from inception to 28 February 2026. The review question was framed using a modified PICOS structure. Because the literature comprised heterogeneous human, translational, and animal studies and was not suitable for pooled quantitative analysis, findings were synthesized narratively, with reporting informed by SWiM principles. Study screening, data extraction, and methodological appraisal were performed by a single reviewer; the review protocol was prespecified but was not prospectively registered. The included studies were grouped into four mechanistic domains: trigger-point microenvironment, fascial biomechanics and tissue gliding, stress-related endocrine-immune and autonomic dysregulation, and central amplification.

**Results:**

The search identified 1,246 records; 318 duplicates were removed, 928 titles and abstracts were screened, and 121 full texts were assessed for eligibility. Fifty-seven studies were included in the qualitative synthesis. Across domains, the literature supports a multilevel account in which the active trigger point constitutes the peripheral nociceptive core; fascial innervation, myofibroblast activity, and impaired hyaluronan-mediated gliding may contribute to local stiffness and persistence; stress-related autonomic and hypothalamic-pituitary-adrenal dysregulation may hinder recovery; and persistent nociceptive input may facilitate central amplification in susceptible phenotypes. Evidence is strongest for local biochemical sensitization and more indirect, though broadly convergent, for sympathetic-fascial and neuroendocrine mechanisms.

**Conclusion:**

Taken together, the available evidence supports a conceptual Stress-Fascia-Pain axis that may help explain recurrence, heterogeneity, and variable treatment response in MPS. This heuristic framework does not displace established trigger-point models; rather, it situates them within a broader integrative account that links local nociception with tissue biomechanics, stress physiology, and pain-system plasticity. Evidence strength varies across domains: it is most direct for trigger-point biochemistry and more indirect or hypothesis-generating for sympathetic-fascial remodeling and endocrine-immune persistence. The framework also supports a multimodal, phenotype-sensitive clinical approach in which local treatment is combined, when indicated, with graded movement, stress regulation, autonomic recovery, sleep restoration, and pain-modulating strategies. Validation through prospective longitudinal and multimodal studies remains a priority.

**Registration:**

The review protocol was specified before screening and data extraction but was not prospectively registered in a public repository.

## 1 Introduction

Myofascial pain syndrome (MPS) remains one of the most common yet most conceptually unsettled problems encountered in musculoskeletal and pain practice (1–3). Clinically, it is associated with myofascial trigger points (MTrPs), palpable taut bands, local and referred pain, restricted movement, and variable functional limitation (4, 5). Even so, its mechanistic framing remains incomplete. Many patients initially present with a localized and mechanically responsive pain problem, but a substantial subgroup later develops recurrent exacerbations, stress-related symptom volatility, disturbed sleep, broader pain sensitivity, and only partial benefit from strictly local treatment (1, 2, 6). That clinical pattern suggests that a focal muscle lesion, although clearly relevant, is unlikely to explain the full course of disease.

The classical integrated hypothesis remains the most firmly established peripheral model for MPS (4, 5). In that framework, abnormal motor endplate activity and excessive acetylcholine release lead to sustained sarcomere contraction, impaired local perfusion, hypoxia, adenosine triphosphate depletion, and progressive tissue acidification. Microdialysis studies have shown that active trigger points have a distinct biochemical milieu, with increased levels of bradykinin, substance P, calcitonin gene-related peptide (CGRP), pro-inflammatory cytokines, serotonin, and norepinephrine (7, 8). This body of work explains why an active trigger point is painful, but it does not fully account for why some cases become persistent, recurrent, or particularly sensitive to stress and poor recovery.

A major gap in the literature is the relative lack of synthesis linking local trigger-point biology with fascia, stress physiology, and nervous-system plasticity. Contemporary fascial research no longer treats fascia as a merely passive wrapping layer. Human and animal work increasingly depicts it as a richly innervated, mechanically responsive, pain-relevant continuum (9–13). At the same time, research in chronic pain and stress biology has shown that psychosocial stress can influence autonomic balance, endocrine signaling, inflammatory regulation, sleep, and pain modulation in ways that may shape the course of persistent musculoskeletal pain (14–17).

Against this background, the present review brings these lines of evidence together within a single conceptual framework, which we refer to as the Stress-Fascia-Pain (SFP) axis. Within this framework, trigger-point biochemistry, stress-responsive fascial mechanics, impaired endocrine-immune resolution, and central amplification are treated as interacting processes rather than rival explanations. The review was conducted using PRISMA 2020 reporting principles and employed structured searches followed by a narrative synthesis suited to a heterogeneous mechanistic literature. The resulting synthesis is primarily integrative and interpretive rather than a conventional systematic review in the narrow sense.

The review had two linked aims. First, it sought to provide a structured synthesis of the mechanistic evidence relevant to the Stress-Fascia-Pain axis in MPS. Second, it sought to judge how convincingly these domains can be connected without overstating causality. Of particular interest was whether the existing literature supports a clinically useful account of persistence: one that explains not only why an active trigger point hurts, but also why some patients remain regionally stiff, physiologically dysregulated, and pain-sensitive long after the initiating mechanical event has passed.

For this reason, we adopted an integrative mapping review rather than a conventional effectiveness review focused on intervention pooling. The relevant literature is methodologically diverse, encompassing microdialysis studies, imaging and biomechanical investigations, fascial histobiology, autonomic and endocrine studies, neuroimaging reports, and mechanistically informative intervention studies. Such material does not lend itself to a single pooled effect estimate, but it can still be reviewed systematically and appraised transparently (18–20). A mapping approach also makes it possible to distinguish areas supported by relatively direct evidence from those that remain more inferential and to identify where future work will require tighter phenotyping and multimodal measurement.

The review was designed to address precisely this conceptual gap. Rather than assuming that one isolated mechanism accounts for all forms of MPS, we examined whether several partially overlapping mechanisms can be arranged into a coherent account of persistence. This distinction matters. A single hierarchical explanation, in which one pathway is presumed to drive every patient and every phase of illness, fits clinical variability poorly. A layered model, by contrast, allows local trigger-point chemistry, fascial mechanics, stress physiology, and central pain processing to contribute in different proportions across patients, anatomical regions, and stages of illness. That view also accords better with clinical experience: some patients improve with local needling or manual therapy, whereas others improve only when sleep, stress load, breathing pattern, pacing, and broader pain regulation are addressed together.

A second unresolved issue concerns anatomical level. Mechanistic discussions of MPS have often concentrated on the contractile unit, the motor endplate, or the tender nodule within muscle. Yet symptoms unfold within a tissue environment that also includes fascia, small vessels, peripheral nerves, immune mediators, and interstitial fluid dynamics. Fascia is particularly relevant because it is not simply contiguous with muscle; it also contributes to force transmission, movement coordination, gliding between tissue layers, and nociceptive signaling (9–13). If fascial tissues become mechanically stiff, biochemically altered, or sympathetically sensitized, they may help sustain pain even when the initial muscular insult is no longer prominent. This broader tissue context may help explain why some patients continue to report pain, tightness, or regional restriction after the most obvious trigger point has been deactivated.

Diagnostic ambiguity remains part of the problem. Although trigger points are familiar to clinicians, operational diagnostic criteria vary considerably across studies, and the reliability of manual palpation depends heavily on examiner training, clinical setting, and body region (6, 21). MPS therefore occupies an uneasy position between a clearly local soft-tissue pain disorder and a broader, stress-sensitive chronic pain phenotype. This ambiguity matters. When the condition is framed too narrowly as an isolated muscle lesion, the contribution of sleep disturbance, stress exposure, autonomic dysregulation, and impaired pain modulation may be underestimated in persistent cases. When it is framed too broadly, the local relevance of trigger-point biology and regional biomechanical dysfunction can be lost. A useful explanatory model therefore has to preserve the local specificity of MTrPs while also accounting for recurrence, spread, and chronicity.

## 2 Methods

We conducted a mechanistic integrative review and reported it using PRISMA 2020 reporting principles (18, 19). A mapping review framework was selected because the review question spans multiple mechanistic domains with heterogeneous study designs, populations, and outcome measures that cannot be meaningfully combined in a pooled quantitative analysis. Unlike a conventional scoping review, which primarily aims to identify the extent and range of available evidence, the present approach also sought to synthesize and critically interpret the mechanistic connections across domains. The review question required integration of heterogeneous evidence drawn from clinical MPS studies, fascia-focused anatomical and histobiological work, stress biology, autonomic physiology, and mechanistic pain neuroscience. Because these literatures differ substantially in design, populations, and outcomes, the goal was not to produce a pooled effect estimate but to synthesize the mechanistic evidence in a structured and transparent way.

The prespecified review question was: how do local trigger-point biology, fascial biomechanics, stress-system dysregulation, and central pain processing together contribute to persistence and recurrence in MPS? We operationalized this question using a modified PICOS framework. Population (P): human participants with MPS, active or latent MTrPs, or closely related myogenous or fascial pain phenotypes when findings were reasonably transferable to MPS; translational tissue and animal studies were retained when they addressed mechanisms that cannot readily be studied directly in humans. Exposure/intervention (I/E): active trigger points, fascial stiffness or impaired gliding, psychosocial or physiological stress, autonomic or endocrine dysregulation, persistent nociceptive input, and related mechanistic perturbations. Comparators (C): healthy controls, non-MPS pain controls, adjacent non-trigger-point tissue, or pre-exposure states where applicable. Outcomes (O): biochemical, electrophysiological, imaging, biomechanical, autonomic, endocrine, inflammatory, or central-sensitization-related findings relevant to persistence or recurrence in MPS. Study design (S): observational studies, mechanistic experiments, imaging studies, translational animal studies, and systematic reviews used for contextual framing.

MEDLINE/PubMed, Scopus, Web of Science Core Collection, and the Cochrane Library were searched from database inception to 28 February 2026. Search strings combined controlled vocabulary and free-text terms related to myofascial pain syndrome, trigger points, fascia, stress, autonomic regulation, HPA-axis signaling, central sensitization, and pain modulation. We also performed backward citation tracking of key reviews and seminal mechanistic papers. Full search strings for each database are provided in Supplementary Appendix 1.

Eligible records included original human studies examining biochemical, electrophysiological, imaging, biomechanical, autonomic, endocrine, or neurophysiological features relevant to MPS or active MTrPs; translational or animal studies with direct mechanistic relevance; and selected systematic, scoping, or narrative reviews used to contextualize domains in which direct MPS-specific evidence was sparse. We excluded conference abstracts lacking sufficient methodological detail, isolated case reports with limited mechanistic value, purely technical intervention papers without biological or phenotypic insight, and studies centered on conditions with no clear transferability to MPS. Given the persistent diagnostic inconsistency across MTrP studies, mechanistic relevance was prioritized over strict adherence to any single historical diagnostic scheme (21).

After deduplication, records were screened by title and abstract, followed by full-text assessment against predefined eligibility criteria. All stages of screening, data extraction, and methodological appraisal were performed by a single reviewer. This represents a methodological limitation compared with conventional systematic reviews, in which duplicate independent screening and extraction are standard; however, it is consistent with the largely interpretive and integrative character of the present synthesis. The review protocol was prespecified before screening and data extraction, but it was not prospectively registered in a public repository (e.g., PROSPERO). Reasons for exclusion at the full-text stage were logged in prespecified categories. Data extraction captured study design, population or tissue model, MPS definition, anatomical site, stress/autonomic/endocrine variables, fascial variables, central sensitization features, principal mechanistic findings, and major limitations. Each included study was mapped to one or more domains of the proposed SFP axis. The PRISMA 2020 flow diagram and the operationalized eligibility criteria are presented in the Supplementary Material (18, 19).

Risk of bias and methodological appraisal were conducted with design-appropriate tools rather than a single universal checklist. Randomized or quasi-randomized studies were considered using RoB 2 principles where applicable; non-randomized clinical studies were appraised using ROBINS-I domains (22); animal studies were assessed using the SYRCLE framework (23); and included systematic reviews were considered with AMSTAR 2 when they materially informed the synthesis (24). For exploratory basic-science and histological studies not well captured by conventional clinical tools, limitations were described narratively with attention to sample representativeness, measurement validity, model relevance, and inferential distance from clinical MPS.

Because the evidence base combined direct clinical studies with translational and basic mechanistic work, a single certainty-grading approach for the entire dataset was not appropriate. Instead, evidence within each domain was characterized as more direct and consistent, supportive but indirect, or hypothesis-generating. The synthesis proceeded in four linked steps: first, the local biochemical changes associated with active trigger points; second, fascial innervation, tissue gliding, and myofibroblast-related stiffness; third, endocrine-immune and autonomic factors that may hinder resolution; and fourth, central amplification and vagal modulation as possible contributors to symptom persistence and variability.

Throughout the review, we adopted a deliberately conservative interpretive stance. The purpose was not to argue that all chronic myofascial pain can be reduced to a single pathway, nor to elevate fascia or stress above the established trigger-point literature. Rather, the aim was to determine whether the available evidence supports a coherent multilevel model of persistence and to identify where conclusions remain provisional because of heterogeneity, indirectness, or weaknesses in study design.

The decision not to perform meta-analysis was methodological rather than simply practical. The included studies varied substantially in case definitions, tissues studied, anatomical sites, measurement platforms, outcomes, and inferential aims. Pooling such material would have created an appearance of precision while obscuring major conceptual non-equivalence. We therefore synthesized the evidence by domain and then examined how those domains might be linked within a broader explanatory account. In keeping with SWiM principles, we made the logic of that synthesis explicit, especially when moving from direct evidence to mechanistic inference (20).

Data extraction was designed to retain mechanistic detail. For each included record, we extracted the pain phenotype or experimental model, sample characteristics, anatomical region, principal mechanistic domain, methods of ascertainment, and the main findings relevant to the proposed SFP axis. We also noted whether findings were best read as direct evidence in MPS, indirect but tissue-relevant evidence, or translational support from adjacent fields. This distinction was important because the literature is unevenly distributed. Trigger-point biochemistry is supported by direct human evidence, whereas the sympathetic-fascial and endocrine-immune dimensions rely more heavily on convergent inference from adjacent human pain states and basic studies.

Because the boundaries of MPS are not defined identically across specialties, some eligibility decisions required explicit judgement. In particular, chronic myogenous temporomandibular pain was considered eligible when the study addressed trigger points, regional muscle pain, stress physiology, autonomic function, or pain modulation in a manner mechanistically relevant to MPS. This decision was made prospectively because the temporomandibular literature contains a disproportionate share of the available endocrine, autonomic, and neuroimaging evidence relevant to persistent myofascial pain. By contrast, studies of generalized nociplastic pain syndromes without a defined myofascial component were not treated as direct evidence for MPS, although they informed interpretation of central amplification and stress intolerance.

The modified PICOS structure reflected the nature of the literature rather than the needs of an intervention meta-analysis. The population of interest comprised adults or adolescents with MPS, myogenous trigger-point pain, or closely related regional myofascial pain states. In human studies, particular weight was given to clinically defined MPS or myofascial temporomandibular pain because those literatures provide the clearest mechanistic measurements. Exposure and intervention domains included trigger-point activity, fascial mechanical properties, stress-related neuroendocrine variables, autonomic measures, nociceptive stimulation paradigms, and treatments informative for mechanism rather than efficacy alone. Comparators included healthy controls, non-MPS pain groups, latent vs. active trigger points, or before-after contrasts within the same participants. Outcomes of interest included biochemical, electrophysiological, imaging, biomechanical, autonomic, endocrine, and pain-processing measures. Eligible study designs therefore encompassed observational clinical studies, experimental physiology studies, translational work, and animal studies when these illuminated biologically plausible pathways relevant to human MPS.

## 3 Results: study selection, study characteristics, and overview of the evidence base

The systematic search identified 1,246 records. After removal of 318 duplicates, 928 titles and abstracts were screened, 125 reports were sought for retrieval, 121 full texts were assessed for eligibility, and 57 studies were included in the qualitative synthesis (Supplementary Figure S1). Full-text exclusions were categorized as wrong condition or population (*n* = 18), wrong study focus (*n* = 16), insufficient mechanistic relevance (*n* = 15), duplicate or overlapping report (*n* = 6), full text unavailable (*n* = 4), and other reasons (*n* = 5). The final evidence base was heterogeneous and not suitable for pooled effect estimation, which is itself informative about the current state of the field.

The included evidence comprised human microdialysis and clinical physiology studies, fascia-focused anatomical and histobiological research, stress, autonomic, and endocrine investigations, translational animal work, and selected review articles used to contextualize domains with limited direct evidence. Methodological appraisal revealed recurring limitations, including inconsistent MTrP definitions, small sample sizes, cross-sectional designs, limited direct applicability of tissue or animal models, and incomplete control of confounding in stress-related studies. Accordingly, the evidence was interpreted as most direct in the trigger-point biochemical domain, supportive but more indirect in the fascial remodeling and endocrine-immune/autonomic domains, and hypothesis-generating for several translational links to central amplification.

Even with these limitations, the evidence showed a recognizable pattern of convergence. Across different methodological traditions, the literature repeatedly pointed to four broad features of persistent myofascial pain: a locally sensitized nociceptive focus, a mechanically relevant extracellular environment, physiological consequences of stress-system dysregulation, and altered central pain modulation in at least a subset of patients. The remainder of the Results section follows that structure, moving from the most direct peripheral evidence toward the more distributed physiological processes that may contribute to chronicity, recurrence, and phenotype variation.

Most included studies were small to moderate in sample size, and several domains relied on cross-sectional comparisons rather than longitudinal designs. Direct causal inference was therefore limited. The risk-of-bias profile also varied by domain. Microdialysis and physiology studies offered strong mechanistic relevance but were often constrained by modest samples and a narrow anatomical focus. Imaging and autonomic studies provided broader systems-level insight, yet many were vulnerable to selection bias, incomplete phenotyping, or residual confounding. Animal and translational studies strengthened biological plausibility but cannot be taken as one-to-one representations of the clinical disorder. This unevenness shaped the synthesis: confidence was highest where human MPS data were direct and replicated, and lower where interpretation depended on extrapolation from related tissues or pain states.

The included studies did not constitute a single homogeneous literature. Rather, they clustered into several partially connected groups. One group consisted of direct human studies examining the local chemistry and physiology of active or latent trigger points, including microdialysis, pressure-pain testing, and experimental provocation paradigms. A second group addressed fascia, connective tissue, or tissue gliding through anatomical dissection, histology, elastography, or broader biomechanical reasoning. A third group examined endocrine, autonomic, or psychophysiological correlates of persistent myofascial pain, often in temporomandibular samples. A fourth group addressed central pain processing through conditioned pain modulation, pressure-pain sensitivity, or neuroimaging. Together, these literatures varied not only in design but also in level of explanation, ranging from molecular events to systems-level physiology.

## 4 The trigger-point microenvironment: the peripheral biochemical core of MPS

Among mechanistic studies in MPS, the microdialysis work of Shah and colleagues remains the clearest human evidence that an active trigger point is a biologically altered lesion rather than merely a palpatory construct (7, 8). These studies demonstrated lower pH and higher concentrations of bradykinin, substance P, CGRP, tumor necrosis factor-α, interleukin-1β, interleukin-6, interleukin-8, serotonin, and norepinephrine in active trigger points than in latent or normal sites. The findings support the view that MPS includes a measurable peripheral nociceptive microenvironment.

These observations indicate that active trigger points are associated with a distinct local biochemical state rather than nonspecific tenderness alone. They also suggest that MPS is not adequately captured by an exclusively structural muscle model or, conversely, by an exclusively central pain model. The peripheral lesion is biologically meaningful, but it is better understood as an ischemic, hypoxic, and inflammatory microenvironment in which nociceptor-sensitizing mediators accumulate and help sustain local dysfunction (7, 8, 22, 23).

**Figure 1:**
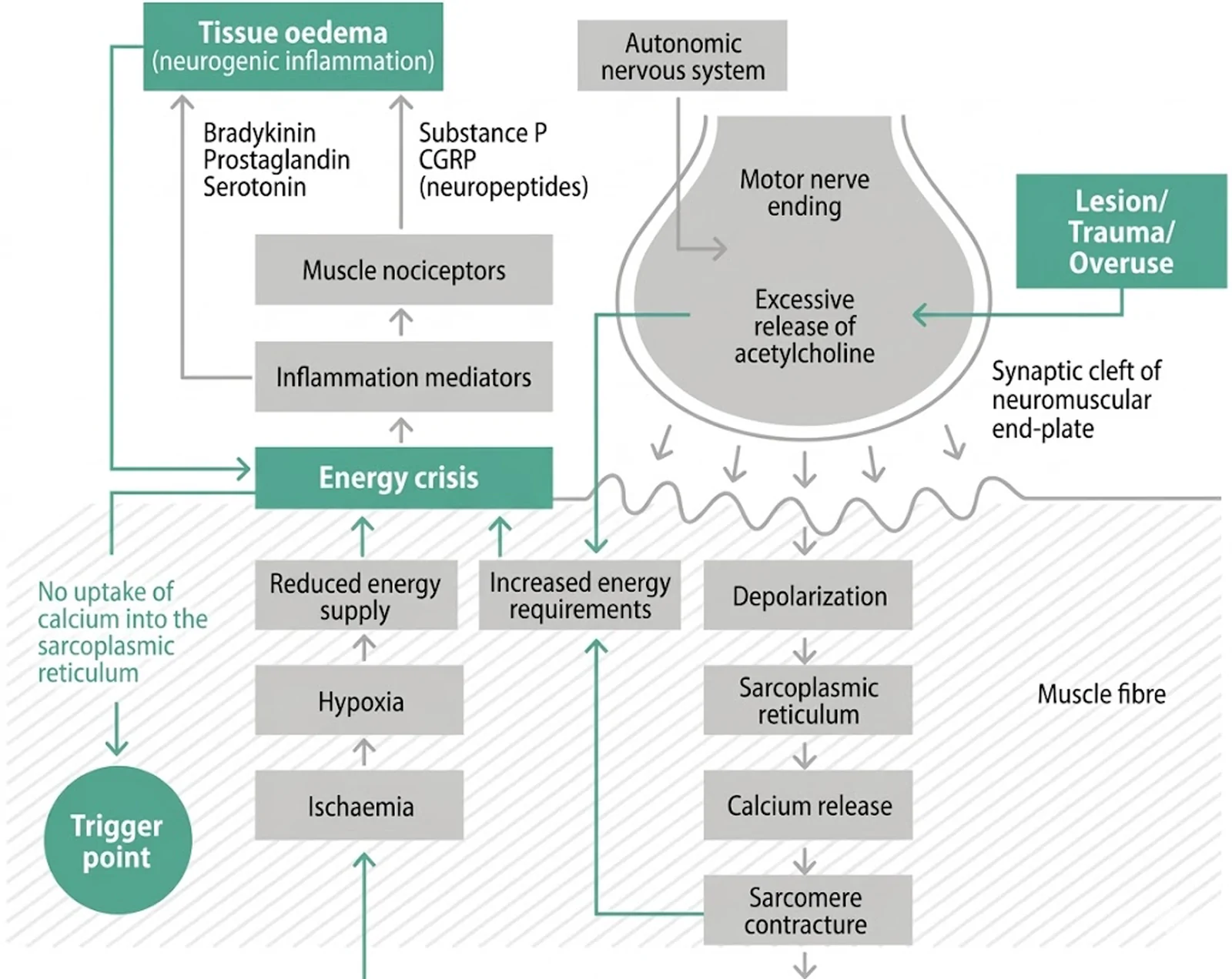
Local ischemia and biochemical sensitization at an active trigger point. Conceptual illustration of the local biochemical environment of an active myofascial trigger point. Local ischemia and hypoxia promote tissue acidosis and the accumulation of nociceptive mediators, including substance P and calcitonin gene-related peptide (CGRP), which may help maintain peripheral pain. This diagram represents a conceptual framework integrating direct human MPS evidence and should not be interpreted as a validated causal pathway.

The observation that biochemical abnormalities may also be present at sites remote from the most symptomatic region suggests that clinically active MPS may extend beyond a single isolated knot (8). This should not be interpreted as evidence of a systemic inflammatory disease, but it does support the idea of regional facilitation or broader nociceptive susceptibility. In that respect, Figure 1 represents the peripheral biochemical starting point of the SFP axis. The active trigger point is best understood as an ischemic and chemically sensitized microenvironment in which reduced pH, neuropeptides, cytokines, and catecholaminergic signaling help maintain local pain and dysfunction.

The trigger-point literature also helps separate direct evidence from the more interpretive layers of the SFP model. Here the evidence is relatively strong and specifically anchored to MPS. For that reason, the active trigger point should remain the starting point of any broader synthesis. Discussion of fascia, autonomic function, endocrine signaling, or central amplification is most convincing when these processes are understood as modifying, sustaining, or generalizing a local nociceptive process rather than replacing it altogether.

At the same time, the microenvironmental account should not be read too narrowly. The available data suggest a continuum rather than a simple binary distinction between healthy tissue and a fully active trigger point. Latent trigger points may already show altered excitability or reduced mechanical threshold even in the absence of spontaneous pain. Clinically, this matters because patients often move between latent and active states in relation to stress load, posture, sleep, repetitive activity, or pain elsewhere. A mechanistic account of MPS therefore needs to explain not only how an active point becomes painful, but also how tissue remains vulnerable to reactivation.

What makes these studies especially important is that they move MPS beyond a purely mechanical description. An active trigger point is not simply a knot that hurts when pressed; it is a metabolically and neurochemically altered microenvironment. Low pH, inflammatory mediators, catecholamines, and neuropeptides provide a plausible substrate for ongoing nociceptor excitation and tenderness (7, 8). They also make sense of everyday clinical observations—why relatively light pressure can reproduce pain, why symptoms flare after overuse or poor sleep, and why an apparently small local lesion can dominate a patient's pain experience.

Evidence summary for Section 4: The trigger-point microenvironmental evidence is the strongest domain in the SFP framework. The biochemical findings (low pH, elevated nociceptive mediators) derive from direct human MPS evidence, principally microdialysis studies in patients with clinically defined active trigger points (7, 8). These findings are replicated across multiple independent investigations and provide the most robust evidence base within this review. Electrophysiological observations (spontaneous endplate activity) also come from direct human studies, although sample sizes are generally modest. The proposed continuum between latent and active trigger-point states is supported by clinical observation and limited experimental comparison but has not been validated longitudinally. Overall, this domain rests on relatively strong direct human MPS evidence, although most studies are cross-sectional, single-site, and small in sample size.

## 5 The sympathetic-fascial axis: molecular mechanisms of stress-induced stiffness

Once the trigger point is recognized as the peripheral biochemical core of MPS, the next mechanistic question is why the surrounding tissue remains tense, mechanically reactive, and prone to recurrence. A purely intramuscular explanation is often insufficient. Fascial tissues contain abundant neural elements, including nociceptive fibers and postganglionic sympathetic fibers, which support the view of fascia as a pain-relevant and autonomically responsive tissue rather than a passive wrapping layer (9, 10).

Direct human evidence that psychological stress, acting through norepinephrine alone, converts fascial fibroblasts into myofibroblasts in clinically defined MPS remains limited. What is better supported is the broader proposition that myofascial tissues can display active contractile behavior and that this behavior is related to myofibroblast density. Histochemical and mechanographic work has shown that fascia is capable of measurable contraction and that contractility correlates with myofibroblast density (12). Mechanical tension is a key regulator of myofibroblast differentiation and persistence, while *α*-smooth muscle actin (*α*-SMA) remains the canonical marker of this contractile phenotype (25, 26).

**Figure 2:**
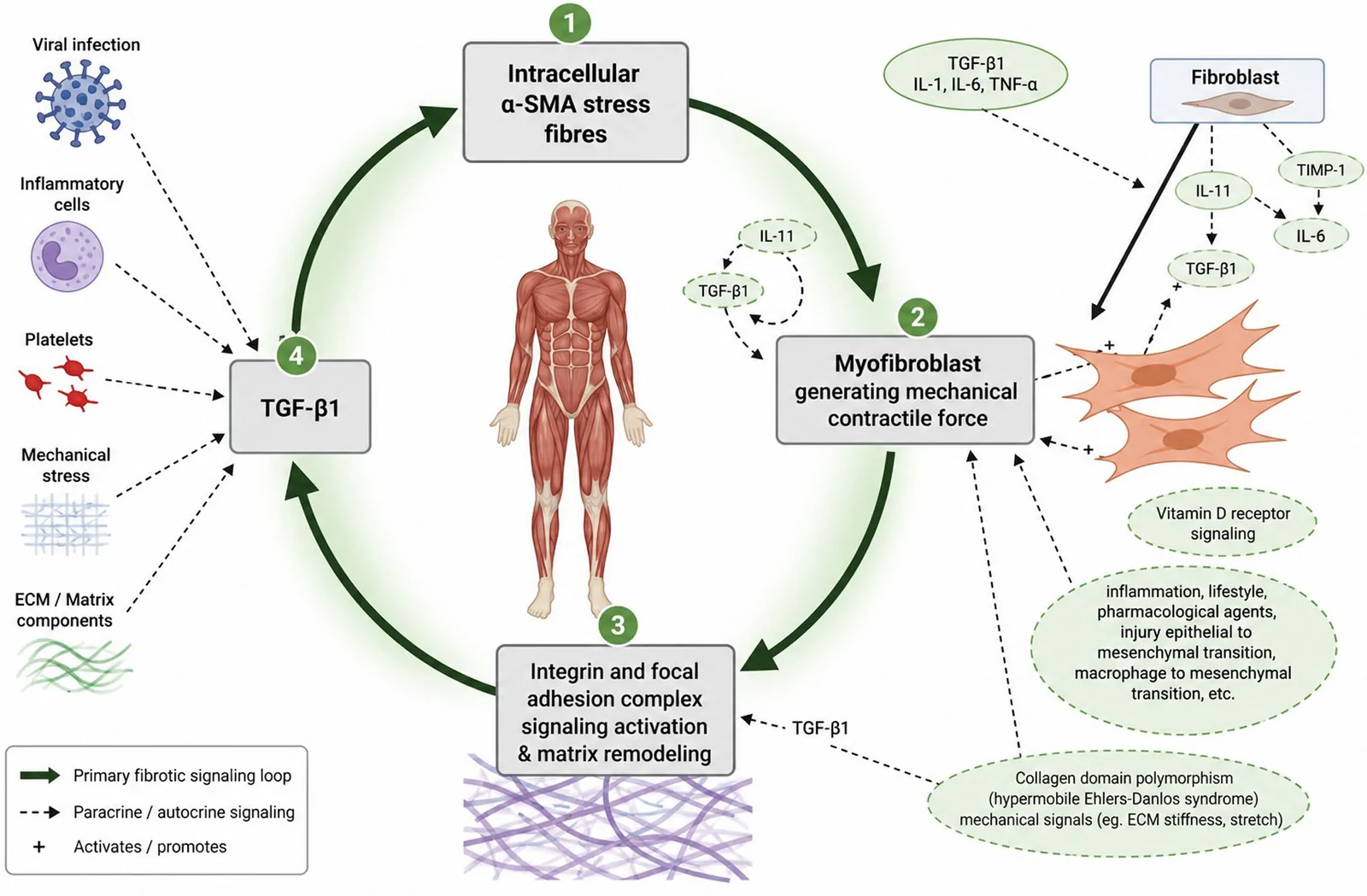
Sympathetic influences, myofibroblast activity, and fascial stiffness. Conceptual illustration of how stress-related norepinephrine release may promote fibroblast-to-myofibroblast transition and *α*-smooth muscle actin (*α*-SMA)-related contractility, thereby increasing fascial stiffness and persistent tissue tension. This diagram represents a heuristic model integrating direct anatomical, translational, and indirect evidence. The complete pathway from psychological stress to persistent fascial remodeling in human MPS remains hypothesis-generating and has not been directly demonstrated in longitudinal clinical studies.

The adrenergic component of this model is more indirect, but it is not irrelevant. In non-musculoskeletal fibroblast systems, norepinephrine can potentiate transforming growth factor-β1 (TGF-β1)-mediated fibrotic signaling and extracellular-matrix production (27). This does not demonstrate the same effect in human fascia in MPS, but it does support the biological plausibility of an adrenergic contribution to fibroplastic change. Figure 2 should therefore be read as a mechanistic interpretation rather than a definitive human causal map.

A second contributor to persistent stiffness is likely to involve altered tissue gliding rather than cellular contractility alone. Deep fascia contains hyaluronan-rich loose connective tissue between fascial layers and between fascia and muscle; this hyaluronan supports smooth sliding between adjacent planes (11, 28). Changes in concentration, molecular organization, pH, and physical environment can increase viscosity and reduce gliding (28). This literature offers a plausible mechanical bridge between microscopic trigger-point chemistry and macroscopic movement restriction. Within this framework, stiffness in MPS may arise from the interaction of at least three layers: trigger-point-related motor and biochemical disturbance, fascial contractile or fibroplastic adaptation, and altered extracellular-matrix gliding. Figure 2 illustrates this mechanically persistent tissue environment.

An additional strength of the sympathetic-fascial framework is that it connects local biomechanics with the lived experience of stress. Patients commonly report worsening symptoms during periods of emotional strain, poor recovery, or sustained vigilance. Such reports are easy to dismiss as secondary or nonspecific. Yet if sympathetic arousal alters fibroblast behavior, muscle tone, blood flow, tissue hydration, or gliding properties even modestly, then stress-related exacerbations become biologically intelligible rather than merely anecdotal. This should not be taken to mean that every flare is psychogenic. It means that psychological and physiological stress may reach the tissue through recognizable biological routes.

Caution is still necessary. Fascial biology has at times been described in overly expansive terms, and the present review does not support the claim that fascia alone explains MPS. The evidence instead suggests that fascial tissues form part of the environment in which trigger points persist. They may amplify local load concentration, reduce tolerance to repetitive strain, and provide a richly innervated substrate through which adrenergic and inflammatory influences can be expressed. Fascia is therefore better understood as a relevant mediator and modifier of pain than as a stand-alone master explanation.

This part of the model is clinically appealing because it may help explain a familiar paradox: many patients report marked tightness, pulling, or tissue restriction even when gross structural damage is absent. If part of that experience reflects altered fascial contractility, densification, impaired sliding, or increased innervation density, then the symptom of “stiffness” is not merely subjective language but a plausible expression of tissue-level dysfunction (9–13). This view also fits the observation that some patients respond to movement-based or manual interventions not because a lesion has been “put back in place,” but because tissue glide, force distribution, and local nociceptive input have changed.

Evidence summary for Section 5: Fascial innervation (including nociceptive and sympathetic fibers) is supported by direct human anatomical evidence (9, 10). Myofibroblast-related fascial contractility is demonstrated in human and animal fascial tissue (12, 25, 26). Hyaluronan-mediated gliding impairment is supported by human cadaveric and *in vivo* imaging evidence (11, 28). However, the proposed pathway linking psychological stress, sympathetic norepinephrine release, fibroblast-to-myofibroblast transition, and persistent fascial stiffness specifically in MPS patients remains largely hypothesis-generating. The adrenergic potentiation of TGF-β1-mediated fibrotic signaling derives from non-musculoskeletal fibroblast systems (27), and its direct applicability to human fascia in MPS has not been demonstrated. Alternative explanations for fascial stiffness—including biomechanical loading, pain-related guarding, movement restriction, and physical inactivity—have not been adequately excluded. This domain should therefore be regarded as biologically plausible but incompletely demonstrated in clinically defined MPS.

## 6 The endocrine-immune pillar: HPA-axis dysregulation and failure of resolution

If Figure 1 depicts the trigger point as a chemically sensitized peripheral focus and Figure 2 shows how stress-responsive fascial mechanics may sustain tissue stiffness, the next question is why this state is not efficiently resolved. In chronic pain more broadly, that question is increasingly framed in terms of stress intolerance, involving dysregulation of the autonomic nervous system and the HPA axis rather than a single uniform endocrine abnormality (14–16). Psychosocial stress, disrupted sleep, pain-related vigilance, and ongoing nociceptive load may together place the organism under sustained physiological strain, thereby weakening processes that would otherwise limit local inflammation and restore tissue homeostasis.

For MPS specifically, direct endocrine evidence remains limited and is drawn disproportionately from chronic myogenous facial pain and temporomandibular myofascial pain phenotypes. Even so, these studies are informative. Experimental stress testing has shown greater cortisol, adrenaline, and noradrenaline responses, as well as slower recovery, in patients with myofascial pain than in healthy controls (29). Other work points to altered negative-feedback regulation rather than to a single reproducible basal cortisol signature (30), and short-term symptom improvement does not necessarily parallel changes in salivary cortisol (31). Taken together, these findings support a cautious conclusion: stress biology in MPS is more plausibly characterized by dysregulated HPA responsivity and feedback than by any single basal cortisol pattern.

**Figure 3:**
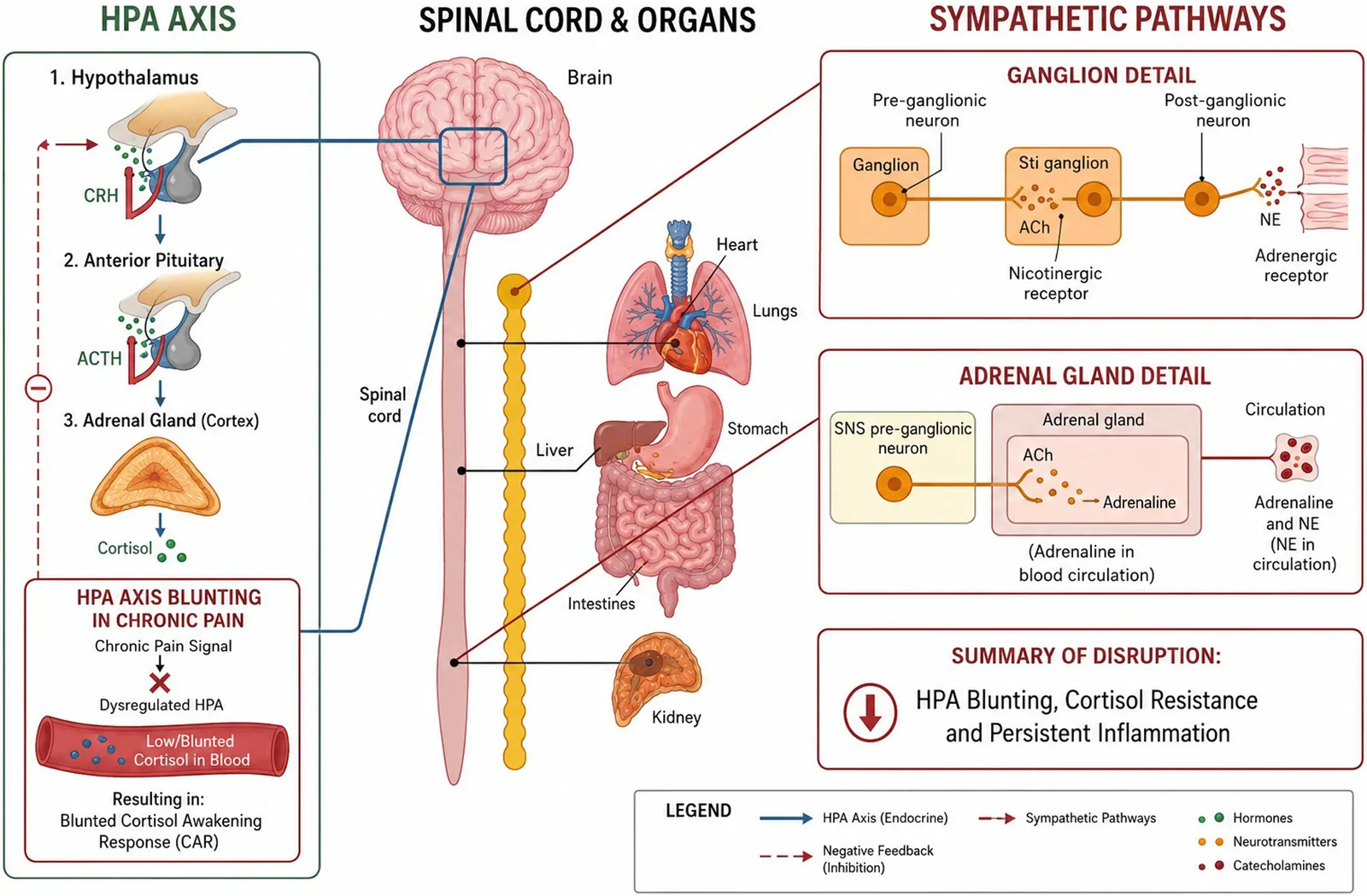
HPA-axis dysregulation and impaired resolution in persistent myofascial pain. Conceptual illustration of how chronic pain may disrupt HPA-axis regulation, alter cortisol responsivity, and reduce endogenous anti-inflammatory control, thereby delaying recovery. This diagram represents a heuristic framework. Direct endocrine evidence in clinically defined MPS is limited and derived disproportionately from temporomandibular pain populations.

The endocrine-immune importance of this dysregulation becomes clearer when glucocorticoid signaling is considered functionally rather than purely quantitatively. Under chronic stress, the anti-inflammatory efficacy of glucocorticoids may be reduced, allowing pro-inflammatory signaling to persist despite ongoing HPA-axis engagement (32, 33). Inflammatory cytokines may in turn impair glucocorticoid-receptor function, creating a feed-forward interaction between inflammation and weakened endogenous restraint (33). Figure 3 should therefore be read as a model of impaired resolution rather than as an argument that all MPS is caused by cortisol deficiency. In this view, persistent myofascial pain may be sustained when local nociceptive inflammation coexists with dysregulated HPA-axis signaling and reduced glucocorticoid-mediated control.

Another strength of the endocrine-immune pillar is that it guards against an overly local interpretation of unsuccessful treatment. When local interventions—such as dry needling or local anesthetic injection—provide only transient relief despite demonstrated short-term efficacy on pain and disability outcomes (34), the explanation is not necessarily that the diagnosis was wrong or that the trigger point was irrelevant. It may instead indicate that the local nociceptive focus is embedded in a physiological context marked by stress intolerance, inflammatory sensitization, or poor autonomic recovery. Under those conditions, recurrence after technically successful local treatment becomes easier to understand.

This perspective also underscores why sleep disturbance deserves greater attention in MPS research. Sleep loss can heighten pain sensitivity, weaken descending inhibition, alter cortisol rhythms, and increase sympathetic tone. Many patients with persistent myofascial pain describe exactly this pattern: pain disrupts sleep, poor sleep impairs recovery, and impaired recovery increases next-day pain reactivity. Although sleep was not the primary focus of most included studies, it is likely to represent a major interface between local tissue irritability and systemic stress physiology. Future MPS studies should therefore treat sleep not as a background variable but as a plausible mechanistic contributor.

The endocrine literature is particularly relevant to persistence because endocrine systems are central to adaptation and recovery. A transient stress response is not inherently pathological; it is part of normal physiology. The problem arises when repeated or poorly resolved stress exposure alters the timing, amplitude, or feedback sensitivity of the HPA axis and related immune pathways (14–16, 29–33). In a patient with ongoing nociceptive input from trigger points and regionally overloaded tissue, such dysregulation may reduce the capacity to return to baseline after physical or emotional provocation. The result may not be a dramatic endocrine abnormality on routine testing, but rather a subtler state of impaired resilience.

Evidence summary for Section 6: Direct endocrine evidence in clinically defined MPS is limited. The available HPA-axis and catecholamine studies are drawn disproportionately from temporomandibular myogenous pain populations (29–31), and their transferability to generalized MPS requires cautious interpretation. The finding of dysregulated HPA responsivity (rather than a single basal cortisol pattern) is supported by these TMD-based studies but has not been replicated in large, prospective MPS cohorts. The proposed mechanism of impaired glucocorticoid-mediated anti-inflammatory control under chronic stress derives primarily from translational and general chronic pain literature (32, 33) rather than from direct MPS studies. The interaction between persistent inflammation and weakened endocrine restraint remains a biologically plausible but incompletely demonstrated mechanism in MPS. Overall, this domain relies on indirect and adjacent-condition evidence and should currently be regarded as hypothesis-generating for MPS specifically*.*

## 7 Central amplification and allostatic persistence

Once the peripheral lesion is established and mechanically sustained, pain in some patients no longer behaves like a purely local nociceptive event. Recent overviews of MPS increasingly describe central sensitization not as a competing explanation that displaces trigger points, but as a process that can arise from persistent peripheral nociceptive input and then contribute to spread, recurrence, and disproportionate symptom burden (1, 6, 13, 17). The clinically important transition, in other words, is from localized trigger-point nociception to a more persistent and amplified pain state.

Experimental work provides an important bridge. Sustained nociceptive mechanical stimulation of a latent MTrP can lower pressure-pain thresholds at remote contralateral sites, indicating that trigger-point input is capable of initiating broader sensitization processes even in healthy participants (35). Clinical evidence from myogenous temporomandibular pain is consistent with this view: systematic review data support lower pressure-pain thresholds in both trigeminal and remote body regions, and temporomandibular MPS frequently coexists with sleep disturbance, distress, and broader sensory amplification (21, 36).

Neuroimaging findings should be interpreted with caution, but they broadly fit this pattern. Chronic myofascial temporomandibular pain has been associated with abnormalities in trigeminal and limbic systems, with pain severity linked to changes in cingulate and insular regions involved in salience, interoception, and affective pain processing (37). At the same time, endogenous inhibitory control appears heterogeneous rather than uniformly impaired. Conditioned pain modulation may be less efficient in some MPS phenotypes, particularly when pain becomes more persistent and widespread, but current evidence does not support a single centrally determined profile (38, 39).

This reciprocal model has practical consequences. Escalating local procedures in a patient with marked sleep disruption, stress reactivity, and widespread hyperalgesia may yield diminishing returns unless broader pain-modulating strategies are introduced as well. Conversely, assuming that all persistent symptoms are purely central risks neglecting active peripheral nociceptive sources that remain treatable. The SFP axis is useful precisely because it avoids that false choice.

The data supporting this transition are not uniform, but they are clinically meaningful. Reduced conditioned pain modulation, broadened pressure-pain sensitivity, and altered activation in pain-processing regions all suggest that at least some chronic myofascial pain states are accompanied by changes in central processing (36–38). Importantly, these findings should not be read as evidence against peripheral pathology. In pain medicine, peripheral input and central amplification are not competing explanations; they often coexist and reinforce one another. In MPS, ongoing trigger-point activity may help drive central sensitization, while centrally amplified processing may increase vigilance, muscle guarding, and perceived tissue threat.

Central amplification is especially important in explaining the transition from a regionally painful syndrome to a more complex and treatment-resistant phenotype. A patient who initially presents with a discrete taut band and predictable referred pain may, over time, develop sleep-related worsening, diffuse mechanical sensitivity, reduced exercise tolerance, and pain that appears disproportionate to local examination findings. This does not mean that the condition has ceased to be myofascial. Rather, the original regional disorder may have become embedded in a nervous system that has learned to amplify nociceptive input and to respond less efficiently to endogenous inhibitory signals (17, 35–38).

Evidence summary for Section 7: The finding that sustained nociceptive stimulation of a latent trigger point can lower remote pressure-pain thresholds is supported by direct experimental human evidence (35). Reduced pressure-pain thresholds in both trigeminal and remote body regions derive primarily from temporomandibular myogenous pain populations (21, 36), and their transferability to generalized MPS should be interpreted with caution given potential differences in neuroanatomical organization and trigeminal-specific processing. Neuroimaging associations with cingulate and insular regions come from chronic myofascial TMD studies (37) and cannot be assumed to apply identically to other regional MPS presentations. Conditioned pain modulation findings are heterogeneous rather than uniformly impaired (38, 39). Overall, the evidence supporting a transition from localized trigger-point nociception to central amplification is supported by converging clinical and experimental observations, but the specific pathways, timing, and patient-level predictors of this transition remain incompletely characterized. This domain is best regarded as biologically plausible with partial direct human support.

## 8 The vagal counter-regulator*y* axis

**Figure 4:**
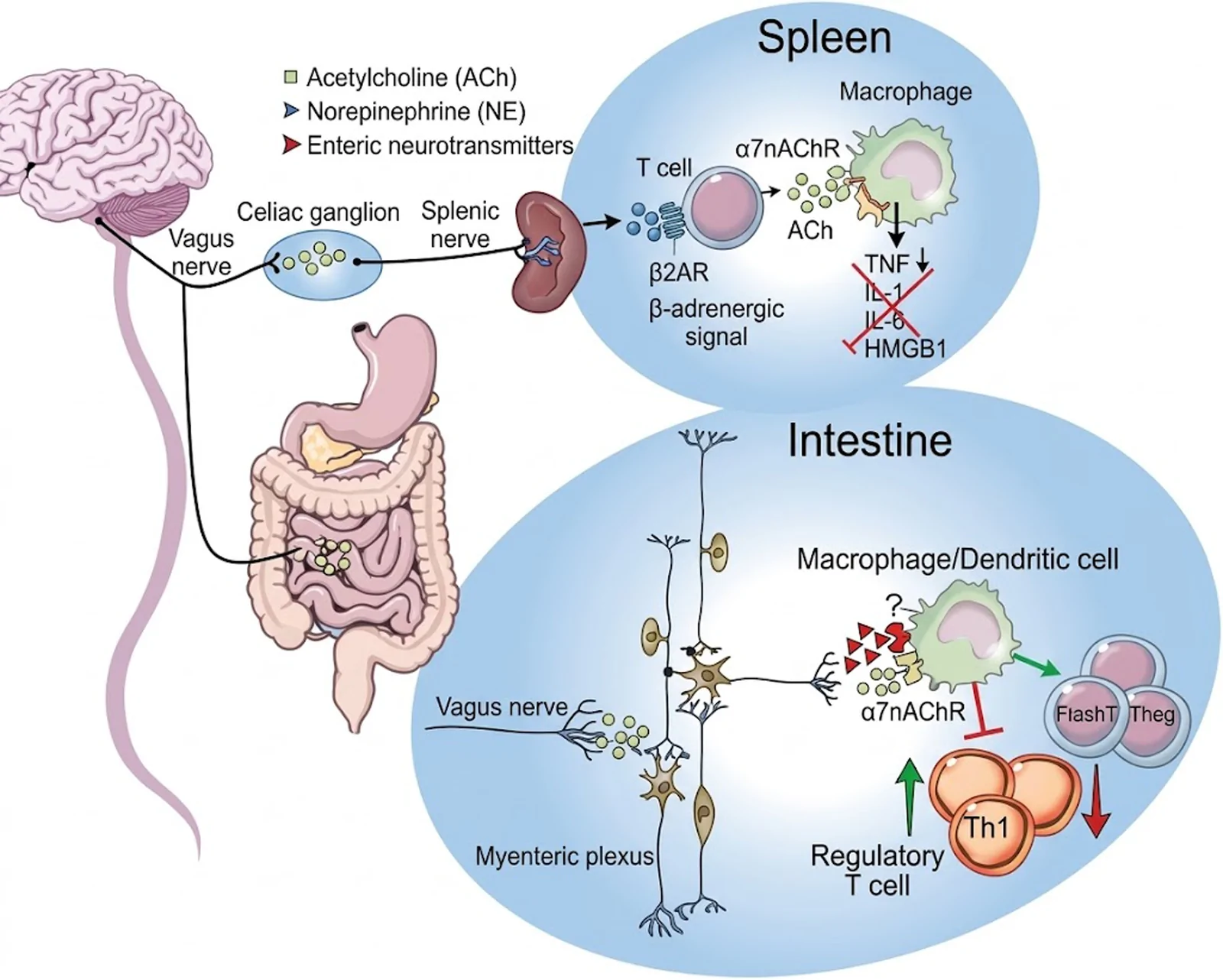
Vagal anti-inflammatory signalling as a potential modulatory pathway. Conceptual illustration of vagal acetylcholine-mediated suppression of pro-inflammatory cytokines such as TNF-α and IL-6 and its possible role as a regulatory pathway within the Stress-Fascia-Pain axis. This diagram represents a hypothesis-generating model. Direct evidence that cholinergic anti-inflammatory signaling normalizes the trigger-point milieu or reverses fascial changes in MPS is currently lacking.

The final component of the SFP axis concerns physiological counter-regulation. If Figures 1–3 depict processes that may contribute to pain persistence, Figure 4 depicts a potential endogenous regulatory pathway: the vagus nerve and the cholinergic anti-inflammatory pathway. The biological basis of this pathway is well established. Vagal signaling can suppress pro-inflammatory cytokine output through acetylcholine-dependent mechanisms, particularly via *α*7 nicotinic acetylcholine receptor-mediated effects on macrophages and related immune cells (40–42).

Caution is required when translating this pathway to the tissue level. Direct evidence that cholinergic anti-inflammatory signaling normalizes the biochemical milieu of active trigger points or reverses fascial densification in clinically defined MPS remains limited. Figure 4 should therefore be understood as a mechanistic model rather than proof of an established MPS-specific therapy. Even so, autonomic data from chronic musculoskeletal pain are compatible with this line of reasoning. Systematic review evidence suggests that adults with chronic musculoskeletal pain often show lower heart-rate variability, consistent with greater sympathetic and reduced parasympathetic modulation (39). Similar patterns have been reported in myofascial temporomandibular pain (40).

This literature also has implications for treatment development. Recent systematic reviews and meta-analyses suggest that non-invasive vagus nerve stimulation may produce a small-to-moderate analgesic effect in some chronic pain conditions, although heterogeneity in diagnosis, stimulation parameters, and study design remains substantial (43, 44). By contrast, evidence specific to low back pain remains insufficient to support routine use (45). Figure 4 should therefore be interpreted as a plausible therapeutic rationale rather than direct proof of efficacy in MPS.

This way of framing the evidence has practical implications. Interventions such as paced breathing, sleep restoration, graded aerobic activity, relaxation-based strategies, and vagally oriented neuromodulation may matter not because they are generic wellness advice, but because they target systems involved in recovery from repeated stress and pain. The early literature on transcutaneous vagus nerve stimulation is encouraging, but still preliminary (43–45). For now, it should be regarded as hypothesis-supporting rather than definitive. Even so, the broader point remains: management of persistent MPS may need to include strategies that actively restore autonomic flexibility, not simply suppress local nociception.

The vagal dimension merits separate attention within the broader autonomic discussion because it directs attention toward recovery rather than dysregulation alone. In many chronic pain conditions, reduced parasympathetic flexibility is associated with poorer sleep, greater symptom volatility, and diminished capacity to down-regulate inflammatory and nociceptive activity (39–44, 46). Direct evidence in MPS remains limited, but the concept is clinically attractive because it offers a biologically plausible counterweight to sympathetic persistence. A patient with recurrent myofascial pain may therefore be understood not only as having excessive nociceptive drive, but also as having insufficient physiological braking. The four mechanistic domains and their clinical implications are summarized in Table 1.

**Table 1:** Mechanistic components of the stress-fascia-pain axis in myofascial pain syndrome.

Domain	Core alteration	Biological mechanism	Clinical implication
Peripheral biochemical core	Active trigger-point microenvironment	Local ischemia, hypoxia, lower pH, and enrichment of nociceptive mediators including substance P, CGRP, bradykinin, cytokines, serotonin, and norepinephrine	Local tenderness, referred pain, mechanical pain reproduction
Fascial biomechanical persistence	Stress-responsive tissue stiffness	Fascial innervation, myofibroblast-related contractility, *α*-SMA expression, altered hyaluronan-mediated gliding, and reduced tissue excursion	Persistent tautness, movement restriction, recurrence after local provocation
Endocrine-immune dysregulation	Failure of biological resolution	Altered HPA-axis responsivity, inefficient glucocorticoid-mediated restraint, and sustained low-grade inflammatory signaling	Stress-linked symptom flares, slower recovery, incomplete resolution
Central amplification	Allostatic pain persistence	Wider nociceptive facilitation, reduced thresholds, temporal summation, and phenotype-level inefficiency of inhibitory modulation	Hyperalgesia, regional spread, disproportionate symptom burden
Autonomic counter-regulation	Vagal/cholinergic brake	Acetylcholine-dependent anti-inflammatory signaling and parasympathetic recovery mechanisms	Potential reduction in inflammatory persistence and autonomic dominance

A substantial proportion of the autonomic, endocrine, psychophysiological, and neurophysiological evidence discussed in support of the proposed Stress-Fascia-Pain framework originates from temporomandibular myogenous pain and temporomandibular disorder (TMD) populations. These studies are valuable and mechanistically informative; however, extrapolation from trigeminal pain disorders to generalized MPS requires careful qualification. TMD and generalized MPS may differ in neuroanatomical organization (trigeminal vs. spinal innervation), autonomic regulation, central pain-processing profiles, and clinical presentation. The trigeminal system has unique features, including a distinct brainstem nucleus, dense cortical representation, and a particularly high density of sensory innervation, which may influence autonomic, endocrine, and neuroimaging findings in ways that do not directly generalize to spinal-level myofascial pain. For these reasons, readers should note that the proposed endocrine-immune and central amplification components of the SFP framework rely substantially on TMD-derived evidence and should interpret these findings as mechanistically informative rather than directly validated for all forms of MPS. Table 2 includes a source population column to help readers distinguish direct MPS evidence from adjacent-condition evidence throughout the review.

**Table 2:** Evidence Hierarchy across mechanistic domains of the stress-fascia-pain axis.

Mechanistic domain	Type of evidence	Source population	Major limitations	Confidence level
Trigger-point biochemistry	Direct human MPS evidence (microdialysis, physiology)	Clinically defined MPS patients with active trigger points	Small samples; cross-sectional; single anatomical site	Relatively strong
Fascial innervation and gliding	Direct human anatomical and imaging evidence	Human cadaveric/biopsy; *in vivo* imaging cohorts	Not MPS-specific; cadaveric tissue limits functional inference	Moderate
Sympathetic-fascial remodeling	Translational/animal; non-musculoskeletal fibroblast models	Non-MPS fibroblast cultures; animal tissue models	No direct human MPS validation; alternative explanations not excluded	Hypothesis-generating
HPA-axis and endocrine-immune dysregulation	Indirect human evidence; adjacent-condition studies	Predominantly TMD myogenous pain; general chronic pain cohorts	TMD-to-MPS transferability uncertain; no large prospective MPS cohorts	Hypothesis-generating
Central amplification	Partial direct human evidence; adjacent-condition neuroimaging	Experimental MPS provocation; TMD neuroimaging; broader chronic MSK pain	Heterogeneous CPM findings; neuroimaging from TMD not MPS; cross-sectional	Moderate
Vagal counter-regulation	Translational; general chronic pain HRV data; early-stage VNS trials	Broader chronic MSK pain; mixed chronic pain; not MPS-specific	No MPS-specific VNS trials; heterogeneous stimulation protocols	Hypothesis-generating

## 9 Discussion

The present review addresses a familiar problem in the MPS literature: although MPS is common in clinical practice, its diagnosis, classification, and mechanisms remain unsettled (1, 2, 6, 21). Our aim was not to discard earlier models, but to place them in relation to one another. Rather than treating trigger-point biochemistry, fascial dysfunction, stress-system dysregulation, and central sensitization as separate or competing accounts, the SFP axis considers them as interacting layers within the same clinical problem.

This interpretation helps explain why some patients present with a relatively localized and mechanically responsive syndrome, whereas others develop recurrent flares, stress-linked symptom volatility, impaired recovery, disturbed sleep, and broader pain sensitivity. It also helps explain why a strictly local intervention may work well in one phenotype yet yield only partial or short-lived benefit in another. In this model, local techniques remain important because the peripheral lesion is real; however, outcomes are likely to improve when local treatment is situated within a broader management strategy that also addresses movement tolerance, autonomic load, sleep, stress reactivity, and pain-related threat processing (1, 2, 6, 15).

Another strength of the model is that it incorporates fascia without assigning fascia more explanatory weight than the evidence can bear. Fascial innervation, myofibroblast-related contractility, and altered gliding offer plausible mechanisms for persistent stiffness and reduced tissue excursion (9–13, 25–28). At the same time, the model does not claim that fascia alone explains MPS. Instead, it treats the muscle-fascia unit as a biologically relevant bridge between the trigger point and the wider pain system.

Several important limitations should be acknowledged. First, the present review represents a largely interpretive synthesis of heterogeneous evidence rather than a conventional systematic review with duplicate screening and independent data extraction. All stages of screening, data extraction, and methodological appraisal were performed by a single reviewer, and the protocol was not prospectively registered. These methodological features limit reproducibility and increase the risk of selection or interpretation bias. Second, the unevenness of the evidence across domains is a fundamental constraint. Trigger-point biochemistry is supported by relatively direct human MPS evidence, whereas endocrine-immune dysregulation, stress-driven fascial remodeling, and vagal counter-regulation rely more heavily on indirect, translational, or adjacent-condition evidence. Third, diagnostic inconsistency remains a major challenge: systematic review evidence shows substantial variation in the criteria used to define MTrPs across studies, which limits comparability (21). Fourth, a substantial proportion of the endocrine, autonomic, and neuroimaging evidence derives from temporomandibular myogenous pain populations, and the transferability of these findings to generalized MPS requires cautious interpretation given differences in neuroanatomical organization and trigeminal-specific processing. Fifth, most included studies are cross-sectional, limiting the ability to establish temporal or causal relationships between proposed mechanistic layers. The absence of longitudinal multimodal studies and prospective mechanistic validation studies represents a critical gap. For these reasons, the endocrine-autonomic and fascia-stress components of the SFP axis should be read as mechanistically credible and clinically informative, but not yet as fully established causal chains. The SFP axis should be regarded as a conceptual and heuristic framework that organizes heterogeneous evidence and generates testable predictions, rather than a validated pathophysiological model.

These limitations also point clearly to the next steps for research. Priority should be given to transparent and reproducible MTrP diagnostic criteria, longitudinal phenotyping that captures stress reactivity, autonomic measures, sleep disturbance, and broader pain sensitivity, and mechanism-informed trials that test layered treatment strategies in stress-reactive or centrally amplified subgroups. The key question is therefore not whether MPS is “peripheral” or “central,” but how peripheral, fascial, autonomic, endocrine, and central mechanisms become linked in different patients.

One conceptual point deserves particular emphasis. The present synthesis does not attempt to dissolve MPS into fibromyalgia, generic chronic stress, or a catch-all model of central sensitization. The diagnosis retains clinical meaning because trigger points, regional pain distribution, and mechanically reproducible symptoms matter. Yet these local signs do not insulate the disorder from system-level influences. A patient can have a genuine trigger point and a dysregulated stress system at the same time. Indeed, the most difficult cases are often those in which both are present. Recognizing that coexistence may help clinicians move beyond polarizing debates and offer patients a more intelligible account of why their pain can feel both local and widespread, mechanical and stress-sensitive, familiar yet hard to settle.

Experimental treatment studies could also do more to test mechanism directly. Rather than comparing interventions only on pain intensity, future trials might examine whether improvement is accompanied by changes in gliding-related stiffness, heart-rate variability, cortisol rhythm, conditioned pain modulation, or movement confidence. If the SFP axis is valid, different interventions may confer benefit through partly distinct routes: local needling may reduce peripheral nociceptive drive (34), movement-based approaches may restore tissue tolerance and gliding, and vagal or stress-regulatory interventions may improve the physiological terrain in which local pain either resolves or recurs. Mechanism-aware trials would therefore move the field beyond the question of whether a treatment works toward the more clinically useful question of for whom, and by what pathway, it works.

Longitudinal designs are especially needed. Many of the proposed links within the SFP axis are biologically plausible but temporally unresolved. It is still unclear whether autonomic imbalance precedes recurrence, whether fascial densification tracks symptom duration, whether endocrine abnormalities normalize when trigger points are effectively treated, and which patients are most likely to transition from regional myofascial pain to a broader nociplastic presentation. Prospective cohort studies beginning in an early, clearly localized phase would be particularly valuable. Such work could identify which signals—local biochemical severity, stress burden, sleep impairment, reduced conditioned pain modulation, or autonomic dysregulation—best predict persistence.

From a methodological standpoint, the review also highlights the need for stronger phenotyping in future studies. Too many reports use broad labels such as myofascial pain, temporomandibular pain, or chronic neck-shoulder pain without clearly stating whether active trigger points were present, whether symptoms were local or more widespread, and whether sleep, stress load, or autonomic variables were measured in parallel. As a result, literatures that should inform one another often remain only loosely connected. Future studies would be more informative if they combined careful clinical examination with multimodal measurement—for example, local pressure-pain sensitivity, elastography or movement analysis, heart-rate variability, sleep metrics, and stress-related biomarkers within the same sample.

The model may also help explain long-standing disagreements in the MPS literature. Authors who focus on trigger-point injection, dry needling, or local muscle physiology are often studying a genuine part of the disorder. Authors who emphasize stress reactivity, central sensitization, or autonomic dysfunction are often studying another genuine part of the same clinical spectrum. These perspectives appear contradictory only if MPS is assumed to be mechanistically uniform. Once persistence is understood as multilevel, the disagreement softens. Different investigators may simply be observing different stages or phenotypes of a disorder that begins locally but does not always remain local.

This perspective also clarifies why treatment sequencing matters. In a predominantly local phenotype, trigger-point directed therapy, load modification, and restoration of normal movement may reasonably take priority. In a persistence-prone phenotype, however, the same local measures may need to be combined early with strategies directed at sleep restoration, stress regulation, pacing, aerobic reconditioning, and pain modulation. This is not an argument for indiscriminate multimodality. It is an argument for matching the breadth of treatment to the apparent breadth of mechanism. The SFP framework is clinically useful not because it prescribes a single protocol, but because it helps clinicians judge when a narrowly local strategy is unlikely to be enough.

A further clinical implication is that MPS may be approached in phenotypes rather than as a single uniform entity. At one end of the spectrum are patients whose symptoms remain largely local: pain is clearly linked to regional overload, palpation reproduces the complaint, and local treatment together with movement correction is often effective. At the other end are patients with recurrent, stress-sensitive, sleep-disrupted pain, regional spread, and signs of impaired pain modulation; notably, major depression has been found in approximately 39% of MPS patients, with a significant correlation between pain severity and depressive symptom burden (47). These patients may still have active trigger points, but those trigger points exist within a broader physiological context. Distinguishing between these phenotypes does not require abandoning the diagnosis of MPS; it requires a more nuanced view of stage, severity, and system involvement.

It is worth noting that the present framework is not the only recent attempt to integrate peripheral tissue mechanisms and central pain processing within a multilevel explanatory model. Wang et al. (48) recently proposed a “mechanics-fascia-cell-brain” axis for understanding manual therapy as a fascia-directed biomechanical intervention in MPS. Their framework focuses primarily on how mechanical loading during manual therapy is transduced through fascial mechanoreceptors, fibroblast activation, and extracellular matrix remodeling pathways (including TGF-β1/Smad2/3 and Piezo1/YAP/TAZ signaling), ultimately modulating central nervous system pain processing via somatosensory afferents. Both models share the recognition that fascia functions not merely as a passive structural scaffold but as a biologically active signaling interface linking peripheral tissue homeostasis and central pain networks. However, the Stress-Fascia-Pain axis proposed here places greater emphasis on autonomic, endocrine, and stress-related mechanisms—including sympathetic-fascial remodeling, HPA-axis dysregulation, and vagal counter-regulation—that may govern whether myofascial pain resolves or persists, whereas the mechanics-fascia-cell-brain axis concentrates on the biomechanical and mechanotransduction pathways through which therapeutic interventions may operate. These complementary perspectives underscore the growing recognition that MPS requires multilevel integrative models, and their convergence on the fascial system as a central mediating structure lends further support to this direction of inquiry.

### 9.1 Evidence strength and conceptual inference

The Stress-Fascia-Pain axis is proposed as a conceptual and heuristic framework integrating heterogeneous evidence rather than as a validated mechanistic pathway. In this subsection, we explicitly distinguish between levels of evidence supporting different components of the model. First, the following are regarded as reasonably established findings supported by direct human MPS evidence: active trigger points are associated with a distinct local biochemical milieu, including reduced pH and elevated concentrations of nociceptive mediators (7, 8); fascial tissue contains nociceptive and sympathetic innervation capable of mediating pain and autonomic responses (9, 10); and sustained nociceptive input from trigger points can induce broadened pressure-pain sensitivity at remote sites (35). Second, the following are regarded as biologically plausible mechanisms supported by convergent but indirect evidence: myofibroblast-related fascial contractility contributes to persistent tissue stiffness (12, 25, 26); hyaluronan-mediated gliding impairment contributes to mechanical restriction (11, 28); reduced conditioned pain modulation accompanies some chronic myofascial pain phenotypes (38, 39); and autonomic imbalance (lower HRV) is associated with chronic musculoskeletal pain states (39, 40). Third, the following are regarded as hypothesis-generating concepts requiring direct validation: sympathetic norepinephrine drives fibroblast-to-myofibroblast transition and persistent fascial remodeling in MPS (27); dysregulated HPA-axis signaling impairs resolution of trigger-point inflammation in MPS (29–33); vagal cholinergic anti-inflammatory signaling directly modulates the trigger-point microenvironment; and the complete SFP axis operates as an integrated pathophysiological cascade in individual patients.

This distinction is important because the narrative integration of diverse evidence can create an impression of greater coherence than is warranted by the underlying data. Many of the proposed connections between domains are inferred from the convergence of findings across different study types, populations, and anatomical levels, rather than directly demonstrated within a single MPS population. The reader should therefore interpret the SFP axis as a working hypothesis that organizes current knowledge and identifies testable predictions, rather than as a confirmed causal model.

### 9.2 Unresolved controversies and limitations of current knowledge

The present synthesis should be read alongside several unresolved controversies and persistent uncertainties that limit the conclusions that can be drawn.

First, the diagnosis and identification of myofascial trigger points remains a source of continuing uncertainty. Variability in examination techniques, inconsistent diagnostic criteria across studies, and concerns regarding inter-rater reliability remain important limitations of the MPS literature and directly influence the interpretation of mechanistic findings (6, 21). Without standardized, reproducible diagnostic procedures, it is difficult to determine whether different studies are investigating the same clinical entity.

Second, although fascial innervation, altered gliding, and mechanobiological mechanisms are increasingly studied, there remains ongoing debate regarding the extent to which fascial abnormalities represent primary drivers of symptoms vs. secondary adaptations to pain, altered movement patterns, or tissue loading. The possibility that observed fascial changes are consequences rather than causes of persistent myofascial pain cannot be excluded on the basis of current cross-sectional evidence.

Third, some studies in the included literature report inconsistent, equivocal, or negative findings. For example, conditioned pain modulation is not uniformly impaired across all MPS phenotypes (38, 39), and HPA-axis findings do not converge on a single reproducible basal cortisol signature (30, 31). A balanced appraisal requires recognizing that the evidence base is not uniformly supportive.

Fourth, many findings discussed throughout this review demonstrate correlations between stress-related physiology, autonomic measures, fascial characteristics, and pain outcomes. However, relatively few studies directly establish causal relationships between these variables. Cross-sectional designs, which constitute the majority of the included evidence, cannot determine whether observed associations reflect causal mechanisms, shared confounders, or reverse causation.

Finally, the principal evidence gaps that currently limit validation of the Stress-Fascia-Pain model include: (a) the absence of longitudinal multimodal studies tracking patients from early localized MPS through potential transition to persistent, stress-sensitive phenotypes; (b) limited direct human evidence for sympathetic-fascial remodeling and endocrine-immune persistence loops specifically in MPS; (c) substantial reliance on adjacent-condition literature, particularly TMD, for endocrine and neuroimaging conclusions; and (d) the absence of prospective mechanistic validation studies testing the individual components and proposed interactions of the SFP axis.

## 10 Conclusion

Myofascial pain syndrome should not be understood solely as a focal muscular abnormality. The available evidence supports a conceptual layered account in which active trigger-point biochemistry forms the peripheral nociceptive core (supported by relatively strong direct human evidence), fascial dysfunction and impaired tissue glide may contribute to mechanical persistence (supported by moderate direct evidence and hypothesis-generating translational data), stress-related endocrine and autonomic dysregulation may delay recovery (supported primarily by indirect and adjacent-condition evidence, particularly from TMD populations), and altered central pain processing may amplify symptoms in susceptible patients (supported by partial direct and convergent indirect evidence). Not every patient will express all of these mechanisms to the same degree, but the convergence of the literature suggests that persistent MPS is best understood as a multilevel process rather than a single isolated lesion. Framed in this way, the Stress-Fascia-Pain axis offers a conceptual and heuristic framework—rather than a validated causal pathway—that may guide both clinical reasoning and future research. Persistent myofascial pain is most likely to improve when local treatment is matched by careful attention to movement, recovery, sleep, stress physiology, and pain-system modulation. Prospective longitudinal studies and multimodal mechanistic validation are needed to test the individual components and proposed interactions of this model before causal conclusions can be drawn.

## Data Availability

The original contributions presented in the study are included in the article/Supplementary Material, further inquiries can be directed to the corresponding author.
