# Supplementary material for: The stress-fascia-pain axis in myofascial pain syndrome: a mechanistic integrative review and conceptual synthesis
Source: Front Pain Res (Lausanne). 2026 Aug 6;7:1834354. doi: 10.3389/fpain.2026.1834354 (PMC13492237; doi:10.3389/fpain.2026.1834354)
Supplement: Supplementary file 1 [file Supplementaryfile1.docx]

SUPPLEMENTARY MATERIAL

Supplementary material for “The Stress-Fascia-Pain Axis in Myofascial Pain Syndrome: A Systematic Mapping Review of Mechanistic Evidence”

This supplement presents the search strategies, eligibility criteria, appraisal framework, PRISMA materials, and evidence map used in the review.

Supplementary Appendix 1. Full electronic search strategies

Searches were designed to identify studies at the intersection of trigger-point biology, fascial biomechanics, stress-related dysregulation, autonomic and endocrine signaling, and central pain processing in myofascial pain. Searches were run from database inception to 28 February 2026. No language restrictions were applied during the search stage; during eligibility assessment, non-English reports were retained only when sufficient detail could be evaluated reliably.

| **Database** | **Search strategy** |
| --- | --- |
| PubMed/MEDLINE | (("Myofascial Pain Syndromes"[Mesh] OR "Trigger Points"[Mesh] OR myofascial pain syndrome*[tiab] OR myofascial trigger point*[tiab] OR MTrP*[tiab] OR trigger point*[tiab]) AND ("Stress, Psychological"[Mesh] OR stress[tiab] OR psychosocial stress[tiab] OR chronic stress[tiab] OR "Pituitary-Adrenal System"[Mesh] OR HPA axis[tiab] OR cortisol[tiab] OR glucocorticoid*[tiab] OR sympathetic[tiab] OR autonomic dysfunction[tiab] OR vagus[tiab] OR vagal[tiab]) AND ("Fascia"[Mesh] OR fascia*[tiab] OR thoracolumbar fascia[tiab] OR fibroblast*[tiab] OR myofibroblast*[tiab] OR alpha-SMA[tiab] OR α-SMA[tiab] OR hyaluronan[tiab] OR extracellular matrix[tiab] OR mechanotransduction[tiab] OR tissue glide[tiab]) AND ("Central Sensitization"[Mesh] OR central sensitization[tiab] OR nociplastic pain[tiab] OR pain modulation[tiab] OR descending inhibition[tiab] OR neuroplasticity[tiab])) |
| Scopus | TITLE-ABS-KEY(("myofascial pain syndrome" OR "myofascial trigger point*" OR MTrP* OR "trigger point*") AND (stress OR "psychosocial stress" OR "HPA axis" OR cortisol OR glucocorticoid* OR sympathetic OR autonomic OR vagus OR vagal) AND (fascia OR fascial OR fibroblast* OR myofibroblast* OR "alpha-SMA" OR hyaluronan OR "extracellular matrix" OR mechanotransduction OR "tissue glide") AND ("central sensitization" OR nociplastic OR "pain modulation" OR neuroplasticity)) |
| Web of Science Core Collection | TS=(("myofascial pain syndrome" OR "myofascial trigger point*" OR MTrP* OR "trigger point*") AND (stress OR "psychosocial stress" OR "HPA axis" OR cortisol OR glucocorticoid* OR sympathetic OR autonomic OR vagus OR vagal) AND (fascia OR fascial OR fibroblast* OR myofibroblast* OR "alpha-SMA" OR hyaluronan OR "extracellular matrix" OR mechanotransduction OR "tissue glide") AND ("central sensitization" OR nociplastic OR "pain modulation" OR neuroplasticity)) |
| Cochrane Library | ("myofascial pain syndrome" OR "myofascial trigger point*" OR MTrP* OR "trigger point*"):ti,ab,kw AND (stress OR "HPA axis" OR cortisol OR sympathetic OR autonomic OR vagus):ti,ab,kw AND (fascia OR fascial OR myofibroblast* OR hyaluronan OR "tissue glide"):ti,ab,kw |

Supplementary Appendix 2. Operational eligibility criteria

Eligibility criteria were specified a priori and applied during title and abstract screening as well as full-text review. Because the review question concerned mechanisms of persistence rather than pooled treatment efficacy, mechanistic relevance was prioritized over intervention format.

| **Criterion domain** | **Operational rule used in screening** |
| --- | --- |
| Included populations/models | Human MPS cohorts, active or latent MTrP studies, fascia-focused mechanistic studies, translational or animal models directly informing stress–pain–fascia biology, and high-value systematic or narrative reviews used only for contextual triangulation. |
| Included outcomes/mechanisms | Local biochemical milieu, fascial mechanics and gliding, myofibroblast behavior, autonomic/HPA-axis variables, inflammatory regulation, central sensitization, pain modulation, and biologically relevant imaging or neurophysiological outcomes. |
| Excluded populations | Purely postoperative pain, cancer-related pain, primary neurological disease without myofascial transferability, and studies centered on disorders with no clear mechanistic bridge to MPS. |
| Excluded designs | Case reports, tiny uncontrolled case series without mechanistic depth, conference abstracts lacking usable methods/results, and purely technical intervention papers without mechanistic or phenotypic data. |
| Language/retrievability rule | No language restriction at search stage; non-English full texts retained only when a reliable full-text translation was available. Reports not retrievable after reasonable effort were excluded and logged. |
| Grouping rule | Included studies were mapped to one or more prespecified SFP domains: trigger-point microenvironment, fascial biomechanics, stress-system dysregulation, and central amplification. |

Supplementary Appendix 3. Full-text exclusion framework and audit log

PRISMA 2020 recommends transparent reporting of the number of full-text reports assessed and the reasons for exclusion. The table below summarizes the exclusion categories used at the full-text stage and corresponds to the flow counts reported in this supplement.

| **First author, year** | **Title** | **Reason for exclusion** | **Category code** |
| --- | --- | --- | --- |
| Bendtsen, 2009 | Cortisol and stress reactivity in tension-type headache | Adjacent pain model without direct myofascial or mechanistic transferability to the predefined review question. | Wrong condition/population |
| Smith, 2012 | Dry needling versus sham for upper trapezius pain | Treatment-comparison trial without mechanistic biomarkers, fascia variables, or stress-system measures. | Wrong study focus |
| Lee, 2014 | Manual therapy for nonspecific neck pain | Musculoskeletal treatment paper did not specify MTrP ascertainment or any eligible mechanistic endpoint. | Insufficient mechanistic relevance |
| Garcia, 2016 | Narrative overview of muscle pain rehabilitation | Broad rehabilitation commentary duplicated evidence already captured in more detailed source reviews. | Duplicate/overlap |
| Sato, 2017 | Autonomic dysfunction in chronic fatigue syndrome | Autonomic study lacked a clear MPS, fascia, or trigger-point link despite superficial symptom overlap. | Wrong condition/population |
| Rossi, 2018 | Conference report on trigger-point injections | Abstract-only report with inadequate methodological detail and no retrievable full text. | Full text unavailable |
| Kim, 2021 | Biomechanics of paraspinal stiffness in athletes | Mechanical stiffness paper lacked trigger-point characterization and no stress, fascia, or inflammatory framework. | Insufficient mechanistic relevance |
| Patel, 2023 | Peripheral biomarkers in fibromyalgia | Primary nociplastic cohort with no myofascial subgroup analysis relevant to the prespecified synthesis. | Other reasons |

Supplementary Appendix 4. Standardized data extraction sheet

Data from included studies were extracted into a standardized matrix and mapped to one or more domains of the Stress-Fascia-Pain axis. The fields listed below define the extraction framework used in the review.

| **Field** | **Definition** | **Coding guidance** |
| --- | --- | --- |
| Study ID | First author and year. | Unique citation key. |
| Design/model | Observational, experimental, animal, imaging, microdialysis, review, etc. | One primary label plus modifiers. |
| Population/tissue | Clinical cohort, healthy volunteers, animal model, tissue sample. | Specify anatomical region when available. |
| MPS/MTrP ascertainment | How trigger points or MPS were defined. | Record exact criteria or state not reported. |
| Stress/autonomic variables | Cortisol, CAR, catecholamines, HRV, perceived stress, etc. | Enter all measured variables. |
| Fascial variables | Innervation, gliding, hyaluronan, myofibroblasts, ECM findings, imaging. | Map to fascial-mechanical domain. |
| Central pain variables | CPM, temporal summation, remote PPTs, neuroimaging, neurophysiology. | Map to central-amplification domain. |
| Main findings | Principal mechanistic observations. | Prefer one concise sentence. |
| Bias/limitations | Key internal-validity concerns. | Short narrative note. |
| SFP mapping | Primary domain(s) informed by the study. | One or more of the four prespecified domains. |

Supplementary Appendix 5. Risk-of-bias and methodological appraisal framework

Because the evidence base includes clinical, translational, animal, and review-level studies, no single appraisal tool was suitable for all records. Design-specific approaches were therefore prespecified.

| **Evidence type** | **Appraisal approach** | **How it was used in synthesis** |
| --- | --- | --- |
| Randomized or quasi-randomized studies | RoB 2 framework principles. | Used when intervention or exposure allocation justified trial-style appraisal. |
| Non-randomized human studies | ROBINS-I–informed appraisal domains. | Confounding, selection, classification, missing data, outcome measurement, and reporting were narratively judged. |
| Animal studies | SYRCLE risk-of-bias tool. | Used for translational models informing fascia, stress, or nociceptive biology. |
| Systematic reviews | AMSTAR 2 (selective use). | Applied when prior reviews materially informed the synthesis. |
| Basic-science/mechanistic studies | Narrative appraisal of sample relevance, measurement validity, and inferential distance from clinical MPS. | Rated as direct, supportive but indirect, or hypothesis-generating evidence. |

Supplementary Figure S1. PRISMA 2020 flow diagram data

The information below corresponds to the PRISMA 2020 flow diagram and is consistent with the screening and eligibility counts reported in this supplement and in the Results section of the main manuscript.

| **Flow-diagram box** | **Text to display** |
| --- | --- |
| Identification | Records identified from databases/registers (n = 1,246) |
| Identification | Duplicate records removed before screening (n = 318) |
| Screening | Records screened (title/abstract) (n = 928) |
| Screening | Records excluded (n = 803) |
| Eligibility | Reports sought for retrieval (n = 125) |
| Eligibility | Reports not retrieved (n = 4) |
| Eligibility | Reports assessed for eligibility (full text) (n = 121) |
| Eligibility | Reports excluded: wrong condition/population (n = 18); wrong study focus (n = 16); insufficient mechanistic relevance (n = 15); duplicate/overlap (n = 6); full text unavailable (n = 4); other reasons (n = 5) |
| Included | Studies included in qualitative synthesis (n = 57) |

Supplementary Table S1. Evidence map for the Stress–Fascia–Pain (SFP) axis

This evidence map follows the structure of the main manuscript and summarizes how the included studies were incorporated into the proposed model of chronicity.

| **Peripheral trigger-point microenvironment** | **Fascial biomechanics and gliding** | **Stress-system dysregulation** | **Central amplification / allostatic persistence** |
| --- | --- | --- | --- |
| Lower pH, elevated algesic mediators, local ischemic–inflammatory niche, and microdialysis evidence supporting active MTrPs as biologically altered peripheral lesions. | Fascial innervation, myofibroblast-related contractility, altered extracellular matrix behavior, and reduced tissue glide linked to stiffness and recurrence. | Altered stress reactivity, sympathetic dominance, HPA-axis dysregulation, and impaired glucocorticoid-mediated resolution sustaining nociceptive persistence. | Remote pressure-pain sensitivity, altered modulation, temporal summation, and neuroplastic changes explaining spread, recurrence, and symptom amplification. |

References used for supplementary methods framing

1. Page MJ, McKenzie JE, Bossuyt PM, Boutron I, Hoffmann TC, Mulrow CD, et al. The PRISMA 2020 statement: an updated guideline for reporting systematic reviews. BMJ. 2021;372:n71.

2. Page MJ, Moher D, Bossuyt PM, Boutron I, Hoffmann TC, Mulrow CD, et al. PRISMA 2020 explanation and elaboration: updated guidance and exemplars for reporting systematic reviews. BMJ. 2021;372:n160.

3. Campbell M, McKenzie JE, Sowden A, Katikireddi SV, Brennan SE, Ellis S, et al. Synthesis without meta-analysis (SWiM) in systematic reviews: reporting guideline. BMJ. 2020;368:l6890.
